# Eukaryotic cell encystation and cancer cell dormancy: is a greater devil veiled in the details of a lesser evil?

**DOI:** 10.7497/j.issn.2095-3941.2014.0028

**Published:** 2015-03

**Authors:** Abdul Mannan Baig, Naveed Ahmed Khan, Farhat Abbas

**Affiliations:** Department of Biological and Biomedical Sciences, Aga Khan University, Karachi 75300, Pakistan

**Keywords:** Cancer cell dormancy, cancer recurrence, encystation, metastasis

## Abstract

Cancer cell dormancy is the main cause of cancer recurrence and failure of therapy as dormant cells evade not only the anticancer drugs but also the host immune system. These dormant cells veil themselves from detection by imaging and/or using biomarkers, which imposes an additional problem in targeting such cells. A similar form of hibernation process known as encystation is studied in detail for pathogenic unicellular eukaryotic microorganisms. By examination using microarray gene expression profiles, immunocytochemistry tools, and siRNAs during the process of encystation, understanding the covert features of cancer cell dormancy as proposed could be possible. This knowledge can be extended to dormant cancer cells to uncover the mechanisms that underlie this ghost, yet dangerous state of human cancers. We propose a strategy to induce dormancy and exit this state by application of knowledge gained from the encystation induction and retrieval processes in pathogenic eukaryotic microorganisms. Given that early detection and characterization of dormant malignant tumor cells is important as a general strategy to monitor and prevent the development of overt metastatic disease, this homology may enable the design of therapies that could either awake the dormant cell from dormancy to make it available for therapies or prolong such a phase to make cancer appear as a chronic disease.

## Cancer cell dormancy and eukaryotic cell encystation

**Figure 1:**
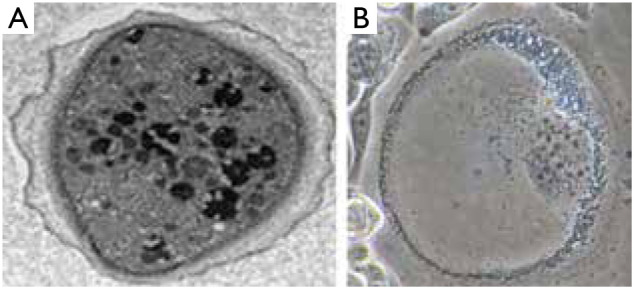
(A) Cyst of *Acanthamoeba castellanii* exhibiting double-walled structure; (B) Induced cancer stem cells in lung carcinoma. During maturation of these cells, mitochondria appeared to play a key role in wall development, as these organelles assumed a centripetal layout helping to delimit border.

In this letter, we bring to debate the thought whether the process of encystation can provide insights into hibernating cancer cells. Cancer cell dormancy and its subsequent recurrence to active form are a daunting threat to our fight against cancer. Determining the epigenetic and metabolic characteristics of these dormant cells would be a significant success in oncotherapy. Given that cancer dormant cells exhibit low metabolic profile and altered epigenetic regulation of gene expression, targeting such ghost cells has become problematic. To date, no specific genetic signature has been identified that could explain the molecular mechanisms associated with tumor dormancy. However, several studies proposed the existence of genetic and molecular pathways that might govern dormancy and escape from dormancy^1^. As evolutionary biology uses unicellular eukaryotic microorganisms to understand the complex mechanisms of multicellular organisms, we hypothesize that studying encystation characteristics in the ancestral “MOTHER” eukaryotic cells, such as primitive eukaryotic pathogenic amoebas, could possibly broaden our understanding of the complexities of cancer cell dormancy. Given that both phenotypes (encystation and dormancy) present a “state of hibernation” (**Figure 1**), the conceptual resemblance is remarkable, but molecular similarities may also exist. We need to determine and examine the stimuli for encystation and dormancy, downstream pathways, effects on organelles, changes in the cell membranes, and metabolic pathways to test this hypothesis. Based on the findings, we can possibly compute the conserved “MOTHER” features in these eukaryotic cell models. Further, examination by using microarray gene expression profiles, immunocytochemistry tools, and siRNAs, we are able to understand the covert features of cancer cell dormancy.

## MOTHER features homology that can facilitate the understanding of cancer cell dormancy

- Maintenance of a receptor-dependent signaling mechanism to initiate hibernation or dormancy.
- Optimizing autophagy pathways that are needed to live in dormancy that dominates over protein anabolic pathways.
- Tweaking biochemical metabolic shift toward bare minimal nutritional requirements needed for survival in a dormant state.
- Hypoxia-resistant survival strategies that help maintain dormancy in an anaerobic state and that can switch on/off when needed.
- Expression of cell surface receptor that could sniff and crosstalk with the microenvironment vascularity and/or nutrients to signal an awakening stimulus from the state of dormancy.
- Reserve nutrient-conserving mechanisms that are needed to be maintained in a dormant state and one that needs minimal space with high caloric value.

## Signaling and crosstalk of cancers in the establishment of tumor dormancy

**Figure 2:**
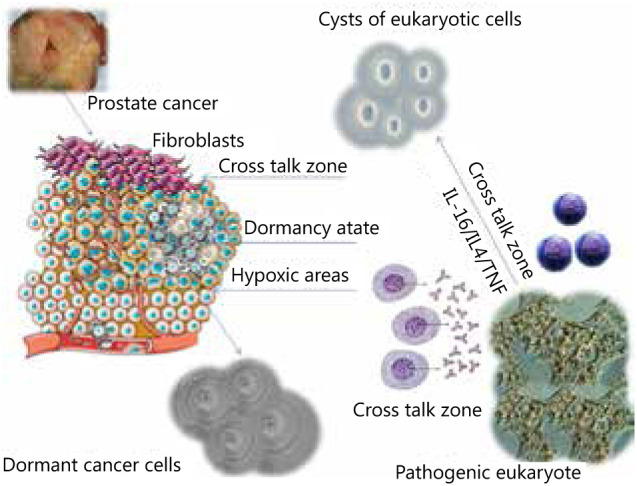
Studying the crosstalks of eukaryotic cells with immune cells and identification of cytokines and/or mediators can help us understand the induction mechanism of microenvironment factors like hypoxia in the causation of dormancy.

The induction of and retrieval from hibernation essentially needs a stimulus from the microenvironment and crosstalk with neighboring stromal cells. Studying the *in vivo* models of eukaryotic cells in areas or organs of the body where they prefer to encyst can reveal cytokines and/or mediators that can help us understand if a similar path is followed by cancer cells in microenvironments that favor dormancy (**Figure 2**). Such knowledge can be used either to wake the cells or induce this state during cancer therapy with drugs and radiation.

**Figure 3:**
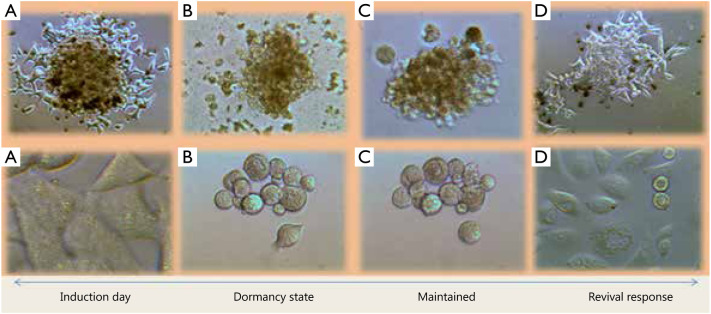
Row of LNCaP (above) and PC3 (below). (A) Induction of dormancy by the same stimuli that provoke encystation in Eukaryotic cells; (B) Cancer cells undergoing dormancy; (C) Dormant state established; (D) Revival from dormancy, by the same stimuli that cause excystation in eukaryotic cells.

Substantial work has been conducted on encystation and excystation in unicellular eukaryotes^2^ like *Acanthamoeba* spp., *Entamoeba*, and *Giardia*. These eukaryotes can be tested for the presence of the aforementioned MOTHER features. Moreover, in-depth studies could be conducted to understand the MOTHER features fully. Recently, the Food and Drug Administration-approved drugs that target cell surface muscarinic (CHRM) G-protein coupled receptors (GPCRs) as antagonists^3^ have prevented these model eukaryotes from initiating the dormant state by blocking cyclic adenosine monophosphate-dependent and calcium-dependent pumps^4^. If tested in an *in vitro* prostate cancer cell model for dormancy, the identical GPCRs antagonists could provide clues to these hibernating mechanisms (**Figure 3**). These findings can be extended to experiments to first induce dormancy and then attempt to awaken cancer cells from that state in animal models. This search of homology in between encystation and dormancy aims to utilize these mechanisms for the two following purposes:

- To awaken and then target cancer cells by chemotherapy and/or radiation;
- To prevent the transformation from dormant state to an active state.

Clinically, the aforementioned attempts would result in a win-win situation. With the former, we stand a chance of a complete cure. With the latter, we observe residual cancer in the form of a chronic disease, but without the fatality associated with the current malignancies. Therapy with luteinizing hormone-releasing hormone in patients with androgen-positive prostate cancer^5^ seems to be an example of such awakening call therapy for dormant cancer cells in this particular form of cancer. Then, these awakened cells can be targeted by anticancer drugs and/or radiation therapy. Deriving a universal awakening stimulus from the examination of the aforementioned homologies in the process of encystation and dormancy could be the Holy Grail for dormancy-related recurrence of cancer.

Molecular biology and biotechnological tools could prove helpful in such homology examinations. Microarray, real-time polymerase chain reaction, and siRNA against the discovered active genes and coupling oligonucleotides with unique receptor-binding ligand could prove useful in the possible eradication of this residual form of cancer. Five decades have passed since the term “dormancy” was introduced^6^, yet our knowledge has barely entered the infancy stage in the understanding of this covert state of a deadly disease. The greater evil seems to hide in the details of a lesser evil. In-depth knowledge of the highlighted “MOTHER” features may open new directions and expose covert targets that could be utilized for therapeutic gains. In particular, *Acanthamoeba* spp. can serve as a useful model for such studies, as its dormancy induction environments and media are well known^4^. To cite few instances of such homologies, the occurrences of features such as encasing within a protective wall^6^ and mucoid shells by dormant cancer cells are apparently similar to the formation of exocyst and endocyst by these pathogenic eukaryote^2^. Mitochondrial metabolic shifts are similar under a state of dormancy^7^ and encystation^4^. We implore to study these homologies to obtain an in-depth understanding of the possible mechanisms that are involved in these processes and extend these mechanisms to dormant cancer cells. In our opinion, these mechanisms could prove a milestone in our endeavor to prevent cancer recurrence and utilize the dormant cancer cell for therapeutic gains.
